# Quantitative susceptibility mapping for iron monitoring of multiple subcortical nuclei in type 2 diabetes mellitus: a systematic review and meta-analysis

**DOI:** 10.3389/fendo.2024.1331831

**Published:** 2024-03-06

**Authors:** Sana Mohammadi, Sadegh Ghaderi, Fatemeh Sayehmiri, Mobina Fathi

**Affiliations:** ^1^ Department of Medical Sciences, School of Medicine, Iran University of Medical Sciences, Tehran, Iran; ^2^ Department of Neuroscience and Addiction Studies, School of Advanced Technologies in Medicine, Tehran University of Medical Sciences, Tehran, Iran; ^3^ Skull Base Research Center, Loghman Hakim Hospital, Shahid Beheshti University of Medical Science, Tehran, Iran; ^4^ Student Research Committee, Faculty of Medicine, Shahid Beheshti University of Medical Sciences, Tehran, Iran

**Keywords:** diabetes, iron, MRI, QSM, basal ganglia

## Abstract

**Introduction:**

Iron accumulation in the brain has been linked to diabetes, but its role in subcortical structures involved in motor and cognitive functions remains unclear. Quantitative susceptibility mapping (QSM) allows the non-invasive quantification of iron deposition in the brain. This systematic review and meta-analysis examined magnetic susceptibility measured by QSM in the subcortical nuclei of patients with type 2 diabetes mellitus (T2DM) compared with controls.

**Methods:**

PubMed, Scopus, and Web of Science databases were systematically searched [following Preferred Reporting Items for Systematic Reviews and Meta-Analyses (PRISMA) guidelines] for studies reporting QSM values in the deep gray matter (DGM) regions of patients with T2DM and controls. Pooled standardized mean differences (SMDs) for susceptibility were calculated using fixed-effects meta-analysis models, and heterogeneity was assessed using I^2^. Sensitivity analyses were conducted, and publication bias was evaluated using Begg’s and Egger’s tests.

**Results:**

Six studies including 192 patients with T2DM and 245 controls were included. This study found a significant increase in iron deposition in the subcortical nuclei of patients with T2DM compared to the control group. The study found moderate increases in the putamen (SMD = 0.53, 95% CI 0.33 to 0.72, p = 0.00) and dentate nucleus (SMD = 0.56, 95% CI 0.27 to 0.85, p = 0.00) but weak associations between increased iron levels in the caudate nucleus (SMD = 0.32, 95% CI 0.13 to 0.52, p = 0.00) and red nucleus (SMD = 0.22, 95% CI 0.00 0.44, p = 0.05). No statistical significance was found for iron deposition alterations in the globus pallidus (SMD = 0.19; 95% CI −0.01 to 0.38; p = 0.06) and substantia nigra (SMD = 0.12, 95% CI −0.10, 0.34, p = 0.29). Sensitivity analysis showed that the findings remained unaffected by individual studies, and consistent increases were observed in multiple subcortical areas.

**Discussion:**

QSM revealed an increase in iron in the DGM/subcortical nuclei in T2DM patients versus controls, particularly in the motor and cognitive nuclei, including the putamen, dentate nucleus, caudate nucleus, and red nucleus. Thus, QSM may serve as a potential biomarker for iron accumulation in T2DM patients. However, further research is needed to validate these findings.

## 1 Introduction

Diabetes mellitus (DM) is a chronic metabolic disorder that is characterized by elevated blood glucose levels (1). Sun et al. (2022) estimated that in 2021, approximately 10.5% (equivalent to 536.6 million individuals) of people aged 20–79 years were affected by diabetes worldwide. This figure is projected to increase to 12.2% (equivalent to 783.2 million individuals) by 2045 (2). In addition to its well-established impact on systemic health, diabetes has been increasingly linked to a wide range of complications affecting various organ systems (3, 4). Among these, the implications of diabetes on neurological health have attracted considerable attention (5). The intricate relationship between diabetes and neurological disorders has sparked extensive research aimed at elucidating the underlying mechanisms and potential biomarkers associated with this complex interaction (6).

Although the impact of diabetes on cognitive function and the development of neurodegenerative conditions has been explored, the assessment of iron accumulation in the subcortical or deep gray matter (DGM) nuclei of the brain, such as the basal ganglia, is relatively uncharted (7–9). Iron, a crucial element in various neurophysiological processes, plays a vital role in brain functions (10). However, excessive iron accumulation or deposition can have detrimental consequences including oxidative stress and neurodegeneration (10–12).

Type 2 diabetes mellitus (T2DM) is a multifaceted metabolic disorder characterized by pancreatic β-cell damage, hyperglycemia, insulin resistance, and insufficient insulin production (8, 13). This condition is intricately associated with cognitive decline, affecting various aspects such as executive function, memory, attention, and visuospatial abilities (8, 14–16), and manifests as structural brain alterations, such as atrophy and iron accumulation (16, 17). The perturbation of insulin signaling exacerbates the distribution of iron in neuronal tissues, resulting in neuronal iron overload and potential complications including neuropathy (18). Furthermore, T2DM is implicated in the genesis of diverse comorbidities and complications including iron deficiency anemia (IDA) (19). Iron metabolism parameters undergo modifications due to the influence of T2DM, leading to changes such as elevated ferritin and hepcidin levels (19). Notably, serum ferritin levels exhibit a discernible association with T2DM (13). Increased hepcidin production in the brain has the potential to impede iron release from macrophages, thereby contributing to iron deposition in the brain (20, 21).

Recent advances in neuroimaging techniques have opened new possibilities for the non-invasive monitoring and quantification of brain iron levels (22, 23). Quantitative susceptibility mapping (QSM) is an advanced magnetic resonance imaging (MRI) technique that is a powerful tool for this purpose (23–25). QSM allows for precise measurement of the magnetic susceptibility (χ) of tissues, including those of the brain (26, 27). It has shown great promise in assessing iron concentrations within various brain structures, offering a potential window into the neurological complications associated with DM (17, 28).

The DGM nuclei and basal ganglia, including the putamen (PUT), globus pallidus (GP), caudate nucleus (CN), red nucleus (RN), substantia nigra (SN), and the largest deep cerebellar cluster of neurons such as the dentate nucleus (DN), which are particularly susceptible to iron overload owing to their high metabolic activity, have become a region of interest (ROI) in diabetes-related neuroimaging studies (29–31). These nuclei play crucial roles in motor control, cognition, and emotion regulation (32, 33). Understanding the implications of iron accumulation in these structures in T2DM patients can provide valuable insights into the neurological manifestations of the disease (17, 28).

Therefore, it is essential to undertake a systematic review and meta-analysis to consolidate the current body of literature on QSM to delineate iron accumulation within DGM structures specific to T2DM. This systematic investigation is critical for assessing the viability of QSM as a potential biomarker for T2DM, shedding light on the depth and distribution of iron deposition. The results of this analysis will contribute significantly to refining our understanding of the intricate relationship between iron dynamics and neurodegenerative processes in T2DM.

## 2 Methods

### 2.1 Search strategy

Preferred Reporting Items for Systematic Reviews and Meta-Analyses (PRISMA) was used (34). The PubMed, Scopus, and Web of Science databases were systematically searched to identify relevant studies published between 2000 and September 2023. The reference lists of eligible studies were manually searched to identify additional relevant publications through a citation search. The search strategy included a combination of terms related to quantitative susceptibility mapping and diabetes mellitus. The following search terms were used: “quantitative susceptibility mapping”, “diabetes mellitus”, and “brain”. The syntax search strategy was adapted for PubMed, Scopus, and Web of Science before searching these databases (**Supplementary Table 1**).

### 2.2 Eligibility criteria

The study developed its inclusion and exclusion criteria and research questions using the Population, Exposure, Comparison, and Outcome (PECO) framework. All studies that assessed changes in magnetic susceptibility in the DGM (Outcome) using QSM (Exposure) in individuals with T2DM (Population) and Controls (Comparison) were eligible for inclusion without any language restrictions. The exclusion criteria included *in vitro* and *in vivo* investigations, books, letters, notes, editorials, surveys, case reports and series, and reviews. Furthermore, studies with findings not related to QSM (utilization of other quantitative MRI techniques, such as R2*) and studies that were not specifically focused on QSM were also excluded.

### 2.3 Screening and study selection

Titles and abstracts were screened by S.G. to identify studies that utilized QSM in T2DM to quantify iron in the DGM nuclei, including the PUT, GP, CN, RN, SN, and DN. S.G. and S.M. independently conducted the selection process, and any disagreements were resolved through discussion. The full texts were screened by two independent reviewers (S.G. and S.M.) to identify the studies that met the eligibility criteria. Any disagreements were resolved through discussion.

### 2.4 Data extraction and quality assessment

Two authors (S. G. and S.M.) collected data extracted from each study. The main data extraction process was organized into several major subdivisions that met the eligibility requirements. The most important factors to note are the characteristics of the study, including the first author’s name and publication year, country of the first author’s affiliation, field strength, coil channels, subjects (patients and controls), DGM nucleus QSM values (formerly noted), and the main findings. The Newcastle–Ottawa scale (NOS) was used by two authors to independently assess quality and evaluate selection, comparison, and outcome biases (35–37). The study was classified from 0 to 9, with a score of 4 indicating a high bias risk, a score of 5–6 indicating a moderate bias risk, and a score of 7 indicating a low bias risk (25).

### 2.5 Meta-analysis

This systematic review and meta-analysis was performed to compare the iron concentrations in patients with T2DM and controls using the QSM method in different regions of the subcortical nuclei using Stata version 17 (Stata Corp, College Station, TX, USA). After data extraction, a meta-analysis was performed to determine whether adequate data were available for a specific region. The standardized mean difference (SMD) between the patient and control groups was used to analyze iron levels. The cut-off values set by Cohen’s d were used to interpret the low, moderate, and high effect sizes (0.2, 0.5, and 0.8, respectively). Analyses were performed using a fixed-effects model. I^2^ statistics were used to assess heterogeneity, and values greater than 50% were considered to indicate moderate to high heterogeneity. Publication bias was visually inspected using funnel plots and quantitatively investigated using Begg’s and Egger’s tests (38, 39). In addition, a sensitivity analysis was performed to assess the effect of excluding each investigation from the combined outcomes.

## 3 Results

### 3.1 Overview of results

**Figure 1:**
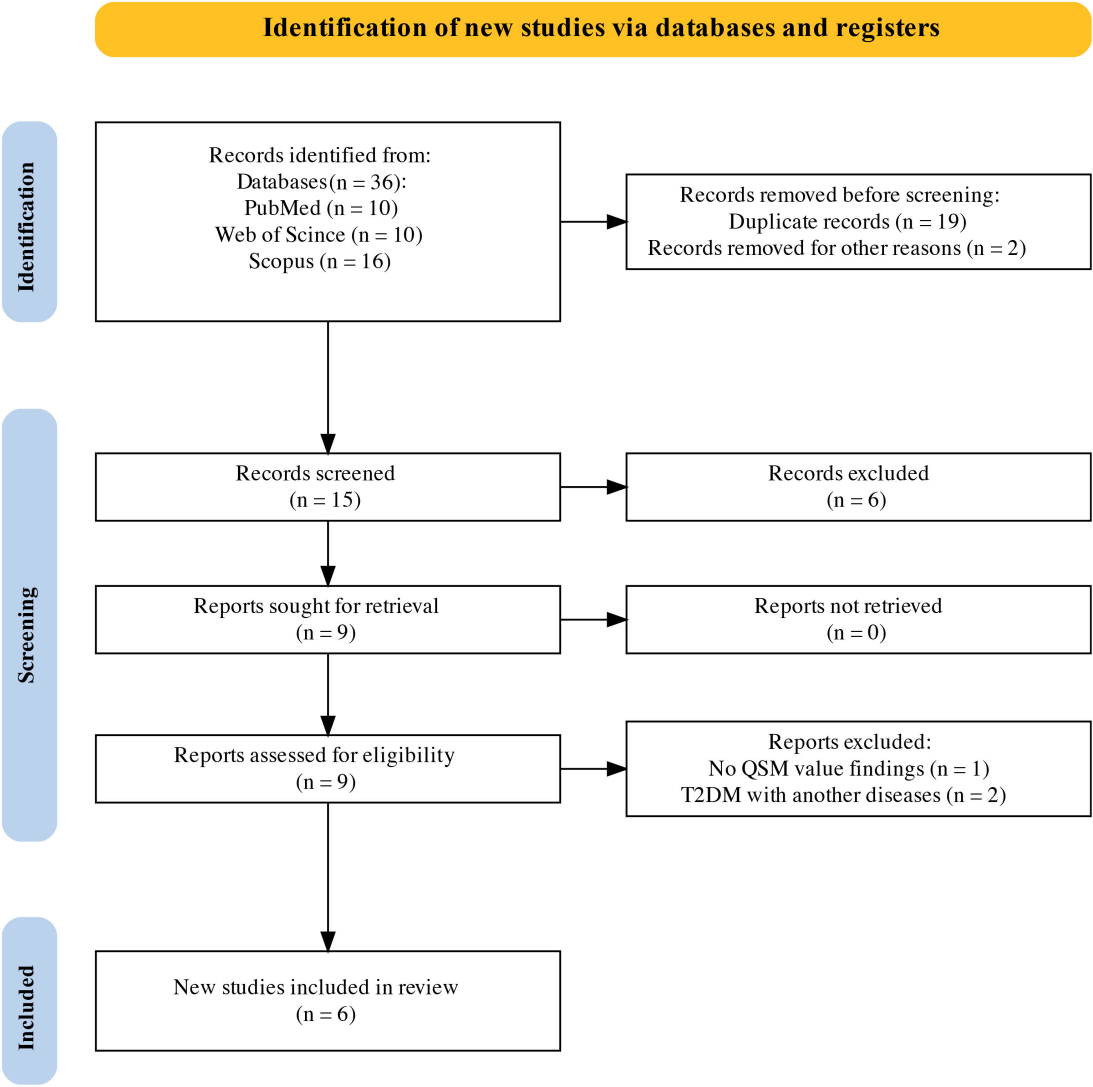
PRISMA flow diagram for systematic review. PRISMA, Preferred Reporting Items for Systematic Reviews and Meta-Analyses.

Six studies including 192 patients with T2DM and 245 controls were included in this systematic review and meta-analysis (**Figure 1**). The basic characteristics and QSM values of the included studies are summarized in **Table 1**. All six studies were cross-sectional in design and published between 2018 and 2023. They were conducted in Asian populations, with five studies from China and one from South Korea. Magnetic field strengths of 3T were used in all studies. QSM values have been reported for various subcortical and DGM nuclei, including the PUT, GP, CN, RN, SN, and DN.

**Table 1:** Study characteristics and QSM values for deep gray matter nuclei in type 2 diabetes mellitus and controls.

First author	Year	Country	FS	Coil	Disease	Patients	Controls	PUT (P) QSM value ± SD	PUT (C) QSM value ± SD	GP (P) QSM value ± SD	GP (C) QSM value ± SD	CN (P) QSM value ± SD	CN (C) QSM value ± SD	RN (P) QSM value ± SD	RN (C) QSM value ± SD	SN (P) QSM value ± SD	SN (C) QSM value ± SD	DN (P) QSM value ± SD	DN (C) QSM value ± SD
**Ni et al., 2023 (** 17)	2023	China	3	8	T2DM	30	27	28.46 ± 11.21	24.98 ± 11.05	113.02 ± 42.46	100.26 ± 48.23	16.42 ± 6.00	12.67 ± 6.34	60.35 ± 27.84	53.06 ± 33.28	113.74 ± 76.2	115.85 ± 79.24	NR	NR
**Hu et al., 2023 (** 28)	2023	China	3	32	T2DM	29	24	114.25 ± 21.165	103.60 ± 8.03	194.15 ± 20.18	182.32 ± 9.44	89.77 ± 11.571	89.90 ± 6.90	178.70 ± 20.05	169.85 ± 16.81	194.38 ± 22.60	186.68 ± 11.192	155.11 ± 16.96	147.34 ± 11.48
**Li et al., 2021 (** 31)	2021	China	3	8 and 32	T2DM	23	82	110.36 ± 23.92	95.54 ± 33.36	183.20 ± 44.64	186.54 ± 48.46	94.02 ± 18.77	80.21 ± 23.18	164.02 ± 37.45	145.53 ± 43.31	166.41 ± 45.93	156.87 ± 38.16	129.10 ± 34.91	106.12 ± 41.18
**Li et al., 2021 (** 40)	2021	China	3	32	T2DM	32	34	52.55 ± 16.86	38.66 ± 19.08	121.72 ± 29.54	109.33 ± 36.03	28.03 ± 10.99	25.17 ± 12.46	168.55 ± 37.11	179.18 ± 36.59	177.43 ± 40.58	179.60 ± 42.89	NR	NR
**Li et al., 2020 (** 8)	2020	China	3	32	T2DM	32	32	121.6 ± 25.37	96.43 ± 25.03	198.78 ± 42.08	196.18 ± 43.51	94.89 ± 44.18	88.29 ± 22.01	179.18 ± 36.59	168.55 ± 37.11	179.93 ± 42.89	177.38 ± 40.58	129.96 ± 33.86	108.02 ± 41.98
**Park et al., 2018 (** 41)	2018	South Korea	3	8	T2DM	46	46	122.95 ± 44.02	114.64 ± 36.95	148.94 ± 55.97	148.22 ± 55.64	96.75 ± 27.89	88.83 ± 26.84	NR	NR	NR	NR	NR	NR

CN, caudate nucleus; DN, dentate nucleus; FS, field strength; GP, globus pallidus; NR, not reported; PUT, putamen; QSM, quantitative susceptibility mapping; RN, red nucleus; SN, substantia nigra; T2DM, in type 2 diabetes.

### 3.2 Meta-analysis and quality assessment results

**Figure 2:**
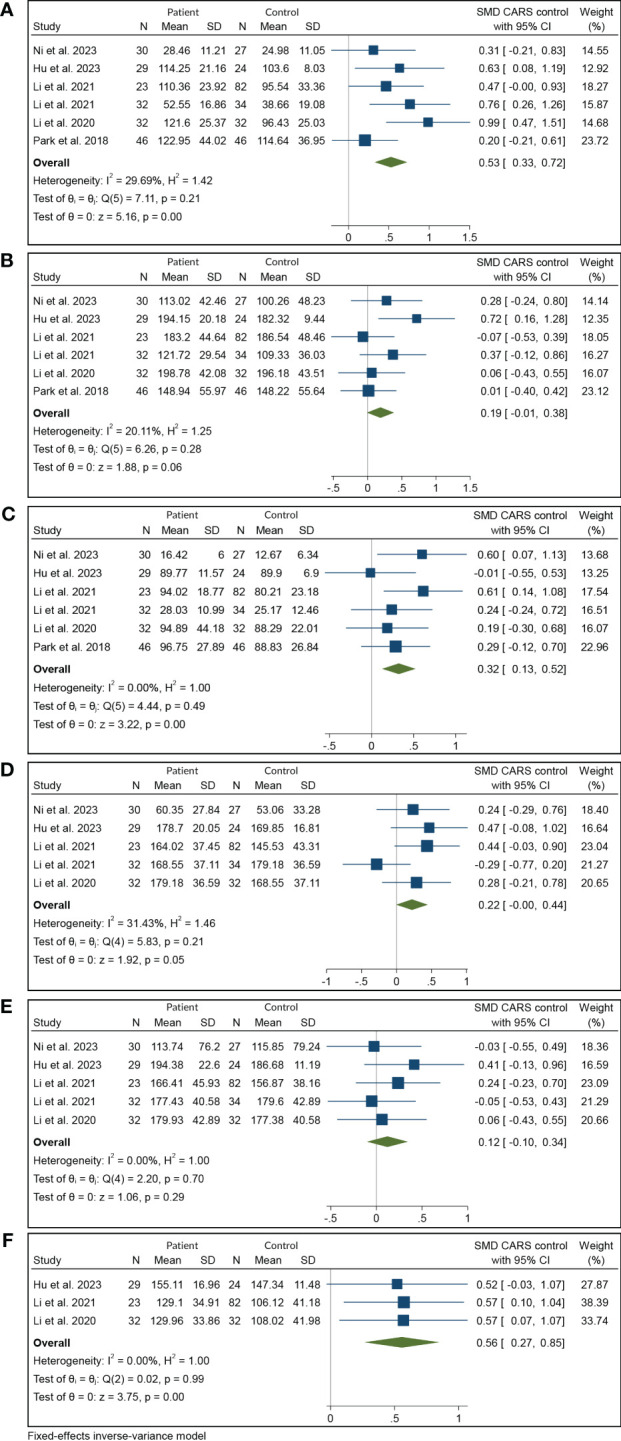
Meta-analysis of QSM values in various subcortical nuclei: **(A)** putamen, **(B)** globus pallidus, **(C)** caudate nucleus, **(D)** red nucleus, **(E)** substantia nigra, and **(F)** dentate nucleus. QSM, quantitative susceptibility mapping.

The systematic review and meta-analysis demonstrated a significant increase in iron deposition, as measured by QSM, in the subcortical nuclei, including the PUT, CN, and DN, in patients with T2DM compared with controls (**Table 2**, **Figure 2**). Specifically, the pooled SMD showed a moderate increase in iron in the PUT (SMD = 0.53, 95% CI 0.33 to 0.72, p = 0.00) and the DN (SMD = 0.56, 95% CI 0.27 to 0.85, p = 0.00) in T2DM patients versus controls. A weak association was found between increased iron levels in the CN (SMD = 0.32, 95% CI 0.13 to 0.52, p = 0.00) and the RN (SMD = 0.22, 95% CI 0.00 to 0.44, p = 0.05) of T2DM patients and controls. Iron deposition alteration in the GP (SMD = 0.19; 95% CI −0.01 to 0.38; p = 0.06) and the SN (SMD = 0.12, 95% CI −0.10, 0.34, p = 0.29) between T2DM patients and controls showed no statistical significance. Heterogeneity ranged from low to moderate in the brain regions. The overall findings remained largely unaffected by any of the individual studies as per the sensitivity analysis (**Supplementary Figure 1**). The methodological quality of the included studies was assessed using NOS scores ranging from 8 to 9 stars, indicating good overall quality of the included studies.

**Table 2:** Meta-analysis results of QSM values in different subcortical nuclei.

Brain region	SMD (95% CI)	p-Value	I^2^ (%)	p-Value of heterogeneity	k
PUT	0.53 (0.33, 0.72)	0.00	29.69%	0.21	6
GP	0.19 (−0.01, 0.38)	0.06	20.11%	0.28	6
CN	0.32 (0.13, 0.52)	0.00	0%	0.49	6
RN	0.22 (0.00, 0.44)	0.05	31.43%	0.21	5
SN	0.12 (−0.10, 0.34)	0.29	0%	0.7	5
DN	0.56 (0.27, 0.85)	0.00	0%	0.99	3

CN, caudate nucleus; DN, dentate nucleus; GP, globus pallidus; PUT, putamen; QSM, quantitative susceptibility mapping; RN, red nucleus; SN, substantia nigra; SMD, standardized mean difference.

Subgroup analysis is commonly used to determine the source of heterogeneity and evaluate differences in effect sizes between subgroups. However, in our study, heterogeneity was inconsiderable. Furthermore, owing to the limited number of studies and the similarity of most characteristics among the included studies, such as geographic distribution, field strength, disease type, magnetic susceptibility measurement method, and methodological quality assessment, subgroup analysis was not deemed necessary for our study.

### 3.3 Publication bias analysis

**Figure 3:**
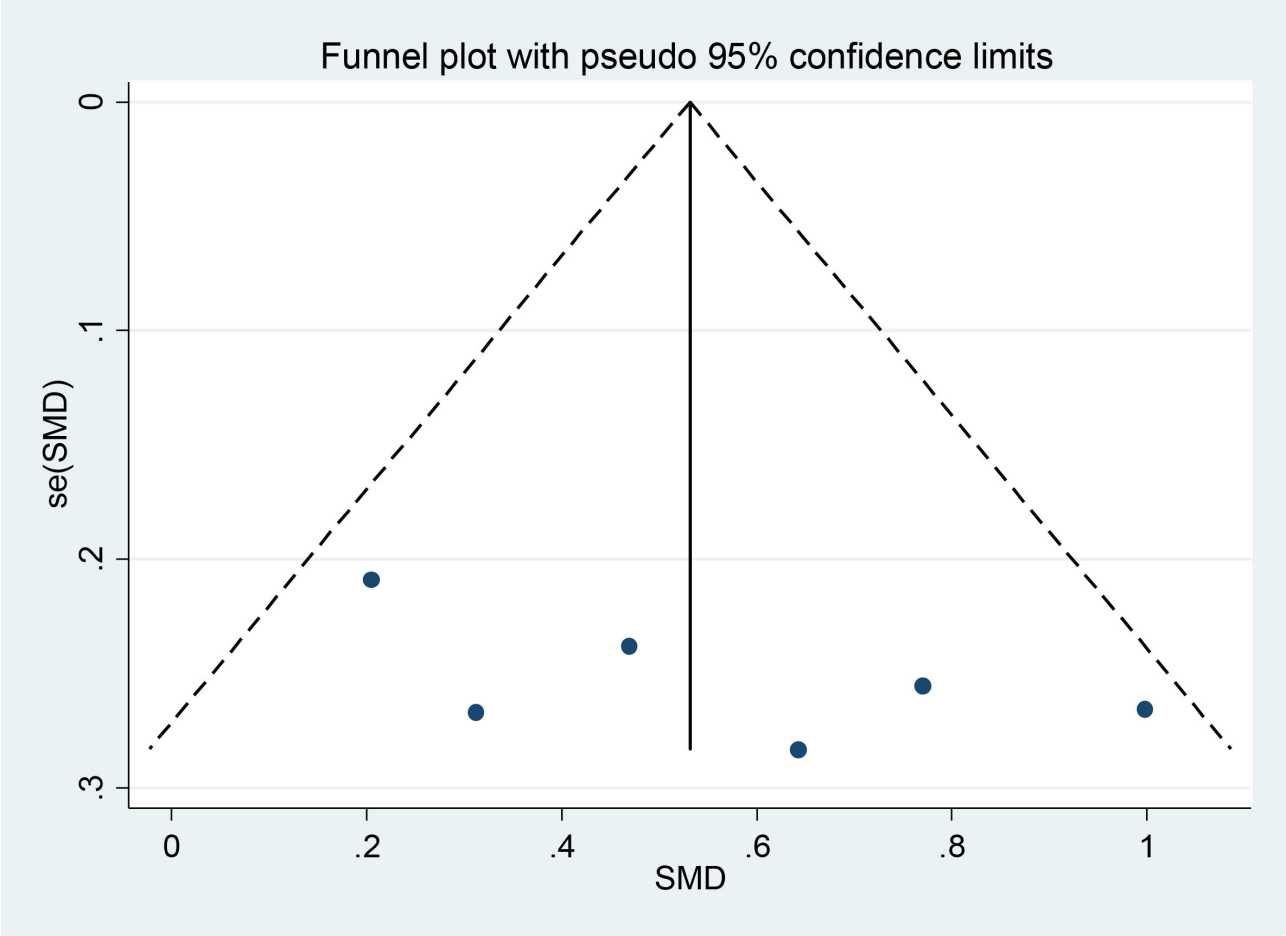
The assessments for publication bias include Begg’s funnel plots.

Begg’s funnel plot in **Figure 3** showed no publication bias (p = 0.348), which was also confirmed by Egger’s test (p = 0.305). The authors used Egger’s funnel plot and Begg’s test to assess the publication bias of the selected articles, where p < 0.05 indicated a significant publication bias. The authors analyzed publication bias using linear regression analysis, which included intercept and slope parameters. This was calculated using the formula yi = a + bxi + ϵi (1) i = 1 ··· r (r = the number of studies), where yi is the standardized estimate, xi is the precision of studies, and ϵi is the error term.

## 4 Discussion

The occurrence and development of T2DM can be directly or indirectly influenced by iron metabolism. Previous evidence has linked diabetes with elevated serum ferritin levels, which is a risk factor for T2DM (21). High iron levels can have negative effects on key aspects of T2DM, such as insulin secretion, insulin resistance, and glucose production in the liver (42). This is true even when iron levels are within the normal range or in cases of excessive accumulation. Although previous evidence has shown that iron overload increases the risk of T2DM and cognitive impairment (16, 42), our meta-analysis revealed that T2DM patients have more iron deposition in the DGM. This may be due to the reciprocal effects of T2DM and iron overload/accumulation (13, 43, 44).

Iron plays a crucial role in metabolic processes and regulation, determining metabolic rates, glucose production, oxygen transport, protein synthesis, fuel utilization, muscle oxygenation, insulin effectiveness, deoxyribonucleic acid (DNA) synthesis, and the characteristics of fat cells (16, 42, 45). Although iron is necessary for neurotransmitter synthesis and mitochondrial function, its excessive accumulation can lead to various diseases, including oxidative stress, diabetic retinopathy, neuronal damage, chronic inflammation, and abnormal glucose and lipid metabolism (10, 16, 46–49).

Previous studies have established a significant link between excessive iron accumulation in the body and the risk of developing T2DM (50). High iron levels can be a risk factor for T2DM and its complications (16, 51–56). Notably, diabetes has also been associated with increased iron accumulation in the brain through multiple mechanisms (16). Elevated levels of iron have been noted in neurodegenerative diseases and disorders (57–60), and excessive iron accumulation could be a significant focus in the treatment of T2DM, as it contributes to and exacerbates damage to the central nervous system (CNS) (16).

Disruption of insulin signaling is one of the primary factors that can lead to impaired iron metabolism and dysregulation of iron homeostasis. Additionally, diabetes-related oxidative stress and inflammation can cause iron release from storage sites and its subsequent accumulation in the brain. These iron accumulations can lead to neurodegenerative diseases such as Alzheimer’s disease (AD), Parkinson’s disease (PD), Huntington’s disease (HD), and amyotrophic lateral sclerosis (ALS) (60–63).

Poor glycemic control can contribute to the development of oxidative stress and inflammation, both of which can disrupt iron balance in neurons and glia (64, 65). Hyperglycemia, in particular, can enhance oxidative stress and inflammation and impair the blood–brain barrier, ultimately leading to excessive accumulation of iron in the brain (66, 67). This accumulation of iron can catalyze the formation of free radicals, which can cause damage to neurons (68, 69). Furthermore, there is a correlation between higher iron levels and impaired cognition in individuals with diabetes (70).

The basal ganglia play a crucial role in working memory, adaptive motor and non-motor functions, executive function tasks, and sensorimotor learning (16, 71–74). Our study offers insights into variations in iron levels across important subcortical nuclei in different regions. In patients with T2DM, abnormalities in iron metabolism may occur in the brain, particularly in the basal ganglia. These abnormalities could potentially contribute to the neurological complications often observed in T2DM patients. Overall, these findings suggest that there is increased deposition of iron in the PUT and DN and to a lesser extent in the CN and RN. These regions play crucial roles in motor control, learning, memory, and cognition through cortico-striato-thalamic circuitry or cortico-striato-thalamocortical neural pathways (8, 16, 17, 28, 31, 40, 41). Degeneration caused by excess iron in these regions likely contributes to the higher prevalence of cognitive decline and movement disorders in patients with T2DM (8, 16, 60, 75). The RN (8, 17, 28, 31, 40) and the DN (28, 31, 40) also play important roles in cognitive and motor functions. Increased iron levels in these nuclei may be related to a higher prevalence of cognitive decline, dementia, and movement disorders in T2DM patients. The neurotoxic effects of iron can lead to degeneration, atrophy, and dysfunction of these structures (17, 31, 40, 62–64).

Moreover, the increase in iron deposition seen in the PUT suggests a potential involvement of iron accumulation in the development of T2DM and age-related iron deposition (16, 31, 40, 63, 75). Iron is known to play a critical role in oxidative stress and neuroinflammation, both of which have been linked to the progression of T2DM (76–78). The greater increase in iron deposition in the DN further supports the idea that iron dysregulation may be related to its important role in cerebellar functions that rely on iron-rich mitochondria (10, 79). The consistent increase in QSM in subcortical regions suggests that abnormal iron accumulation may occur in T2DM, possibly due to mechanisms such as neuronal damage from high blood glucose levels, impaired blood–brain barrier function, inflammation, and microvascular pathologies (8, 17, 28, 31, 40, 41, 62, 63).

Finally, despite the variability across brain regions, consistent findings of increased iron deposition in multiple subcortical areas, such as the main basal ganglia nuclei, support the overall conclusion that QSM could be a useful biomarker for monitoring iron dysregulation in T2DM. To sum up, this meta-analysis helps confirm QSM as a promising biomarker for detecting subcortical iron abnormalities in T2DM (8, 17, 28, 31, 40, 41). QSM offers several advantages over other MRI techniques, such as susceptibility weighted imaging (SWI), for quantifying iron because of its specificity for iron and insensitivity to confounding factors (17, 61). The consistency of QSM findings across studies supports its utility in multi-center studies (80). Longitudinal measurements could help to characterize iron deposition dynamics in diabetes and their relationship with cognitive outcomes.

## 5 Limitations and recommendations

The small number of studies and patients is a significant limitation. To allow subgroup analyses based on diabetes duration, control status, and cognition, larger sample sizes are necessary. Additionally, all studies analyzed in this research were conducted in Southeast Asian countries, with five from China and one from South Korea. This can be attributed to the high prevalence and large population of individuals with diabetes in these areas. China has the highest number of people with diabetes, with estimates of over 140 million in 2021, which is projected to reach 174 million by 2045 (33). The role of researchers in conducting these studies and paying attention to these cases is crucial because of the high incidence of diabetes in these regions. Moreover, the findings of this study highlight the importance of paying attention to the prevalence and occurrence of T2DM as well as the potential brain effects caused by the deposition of substances such as iron. The use of advanced techniques such as QSM to better understand these effects is important and promising.

These findings have important implications for both research and clinical practice. Further investigation is warranted to understand the underlying mechanisms and potential consequences of the observed increase in iron deposition in T2DM patients. In recent years, combining QSM with other MR neuroimaging techniques, such as diffusion-based imaging and functional MRI (17), has allowed for a more accurate examination of alterations in both iron content and myelin density within white matter regions. Such multimodal approaches may provide better information on the interaction between iron dysregulation and myelin disruption or the simultaneous assessment of iron distribution and functional connectivity in T2DM patients. Moreover, integrating QSM with detailed neuropsychological tests in future studies could better define the relationship between regional brain iron levels and neurological complications in T2DM patients.

## 6 Conclusions

This study suggests that patients with T2DM have higher levels of iron in specific brain regions than the controls. This increased iron deposition was more observed in the PUT and DN and less in the CN and RN. These findings indicate that QSM may serve as a potential biomarker for iron accumulation in T2DM patients. Further research with larger sample sizes, longitudinal designs, and multimodal imaging approaches is warranted to validate the role of QSM in assessing iron accumulation and its impact on neurological outcomes in T2DM patients.

## Data availability statement

The original contributions presented in the study are included in the article/**Supplementary Material**. Further inquiries can be directed to the corresponding author.

## Author contributions

SM: Conceptualization, Data curation, Formal analysis, Investigation, Methodology, Project administration, Resources, Software, Visualization, Writing – original draft, Writing – review & editing. SG: Conceptualization, Data curation, Formal analysis, Investigation, Methodology, Project administration, Resources, Software, Visualization, Writing – original draft, Writing – review & editing, Supervision, Validation. FS: Formal analysis, Methodology, Project administration, Software, Supervision, Validation, Visualization, Writing – original draft, Writing – review & editing. MF: Data curation, Formal analysis, Software, Writing – original draft.
