# Supplementary material for: Quantitative susceptibility mapping for iron monitoring of multiple subcortical nuclei in type 2 diabetes mellitus: a systematic review and meta-analysis
Source: Front Endocrinol (Lausanne). 2024 Mar 6;15:1331831. doi: 10.3389/fendo.2024.1331831 (PMC10950952; doi:10.3389/fendo.2024.1331831)
Supplement: Supplementary file 1 [file DataSheet_1.docx]

| **Supplementary Table 1.** Search results on PubMed, Scopus, and Web of Science databases for included studies | | | |
| --- | --- | --- | --- |
| **Database** | **Terms** | **Search Keywords** | **Number of initial record findings** |
| **PubMed** | All Field | ("Quantitative susceptibility Mapping" OR "QSM") AND ("Diabetes" OR "Diabetes mellitus") AND ("Brain") | 10 |
| **Scopus** | Title/Abstract | TITLE-ABS-KEY (“Quantitative susceptibility Mapping" OR "QSM”) AND TITLE-ABS-KEY (“diabetes" OR "diabetes mellitus”) AND TITLE-ABS-KEY (“Brain”) | 16 |
| **Web of Science** | All Field | "Diabetes mellitus" (All Fields) and "Diabetes" (All Fields) and "Quantitative susceptibility Mapping" (All Fields) and "Brain" (All Fields) | 10 |


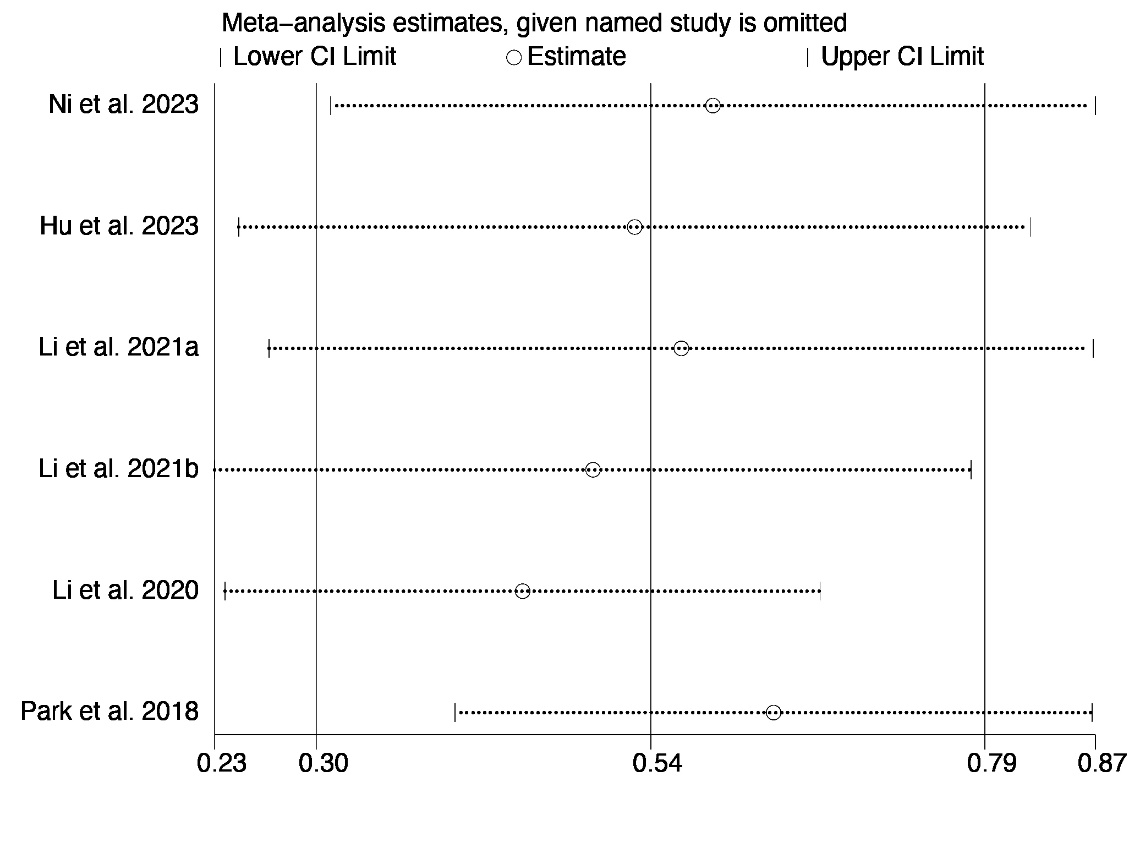


**Supplementary Figure 1.** Sensitivity analysis of included studies
